# Conceptual and practical foundations of patient engagement in research at the patient-centered outcomes research institute

**DOI:** 10.1007/s11136-014-0893-3

**Published:** 2015-01-06

**Authors:** Lori Frank, Laura Forsythe, Lauren Ellis, Suzanne Schrandt, Sue Sheridan, Jason Gerson, Kristen Konopka, Sarah Daugherty

**Affiliations:** 1 Patient-Centered Outcomes Research Institute (PCORI), 1828 L Street NW 9th Floor, Washington, DC 20036 USA; 2 Department of Health Policy and Management, Johns Hopkins Bloomberg School of Public Health and Johns Hopkins Berman Institute of Bioethics, 1809 Ashland Ave, Deering Hall, Baltimore, MD 21205 USA

**Keywords:** Patient engagement, Stakeholder engagement, Patient-centered outcomes research, Comparative effectiveness research, Research funding

## Abstract

**Purpose:**

To provide an overview of PCORI’s approach to engagement in research.

**Methods:**

The Patient-Centered Outcomes Research Institute (PCORI) was established in 2010 to fund patient-centered comparative effectiveness research. Requirements for research funding from PCORI include meaningful engagement of patients and other stakeholders in the research. PCORI’s approach to engagement in research is guided by a conceptual model of patient-centered outcomes research (PCOR), that provides a structure for understanding engagement in research.

**Results:**

To understand and improve engagement in research PCORI is learning from awardees and other stakeholders. Those efforts are described along with PCORI’s capacity building and guidance to awardees via the Engagement Rubric. PCORI’s unique model of engaging patients and other stakeholders in merit review of funding applications is also described. Additional support for learning about engagement in research is provided through specific research funding and through PCORI’s major infrastructure initiative, PCORnet.

**Conclusion:**

PCORI requires engagement of stakeholders in the research it funds. In addition PCORI engages stakeholders in activities including review of funding applications and establishment of CER research infrastructure through PCORnet. The comprehensive approach to engagement is being evaluated to help guide the field toward promising practices in research engagement.

## Introduction

The Patient-Centered Outcomes Research Institute (PCORI) was established as part of the Patient Protection and Affordable Care Act (2010) to fund patient-centered comparative effectiveness research (CER). PCORI requires that the research it funds addresses questions that are important to patients and other stakeholders and measures outcomes that patients and other stakeholders find meaningful. PCORI funding also requires that patients and other stakeholders be actively engaged in the conduct of the research.

In the USA, a substantial and robust research literature based on community-based participatory research (CBPR) models has grown over the last two decades [1, 2] and indicates positive impacts of CBPR. Federal health agencies are also supporting patient engagement in research. The Agency for Healthcare Research and Quality engages patients in the identification of research topics, questions, and outcomes [3]. The National Institutes of Health has a history of working with patients and advocacy organizations to set research priorities [4]. In the United Kingdom, patient involvement in research agenda setting and research activities are increasingly prominent [5]. PCORI’s requirement for patient and other stakeholder engagement in research (referenced here as “patient engagement”) and the legislative mandate to fund new research have injected substantial energy into engaged research in the USA.

Below we present PCORI’s conceptual model for patient-centered outcomes research. We then summarize PCORI’s funding requirements involving engagement of patients and other stakeholders in research, and review efforts to understand engagement in research among awardees and among patients and clinicians. We outline PCORI’s additional activities related to patient engagement in research: inclusion of stakeholders in merit review, data collection from awardees about engagement experiences; and development of the Engagement Rubric that guides engagement at PCORI. Finally, we outline specific funding efforts designed to enhance not just the practice of engagement in research but also understanding of the impact of engagement in research on production of evidence and subsequent uptake of research results. Together these pieces provide the current PCORI view of patient engagement in research along with learnings to date.

## The role of engagement in achieving patient-centeredness in research

For PCORI, ensuring patient-centeredness in research is required in research it funds, and in PCORI’s view, necessitates some form of engagement with patients [6, 7]. The PCORI Methodology Standards include the following: Engage people representing the population of interest and other relevant stakeholders in ways that are appropriate and necessary in a given research context (http://www.pcori.org/assets/2013/11/PCORI-Methodology-Report-Appendix-A.pdf). *Stakeholders* who are the intended end users of research results are expected to participate in the research which can include designing the study, selecting measures, enhancing subject recruitment, interpreting findings, and/or disseminating study findings. Engagement is not an end unto itself and is only one of several strategies for assuring that research, and its results are patient-centered, relevant to the intended users of the research findings, and that the findings can be effectively disseminated. Engagement can take many different forms. The ways in which research partners are engaged may vary by phase of the research, as may the number and type of specific partners who join research projects. For PCORI requirements engagement must meet the goal of active incorporation of perspectives beyond those of the researchers, to inform decisions about research questions, study design, measures used, practical aspects of study implementation particularly related to recruitment and data collection, data interpretation, and/or dissemination of results.

## Conceptual model for patient-centered outcomes research

The conceptual model presented here identifies PCOR concepts and relationships between them and describes the role of engagement in research in PCOR. This model is intended to provide the basis for subsequent evaluative frameworks, to guide evaluation of PCOR, and to serve as a foundation for measurement models, to allow testing of hypothesized relationships between elements in the model.

**Fig 1:**
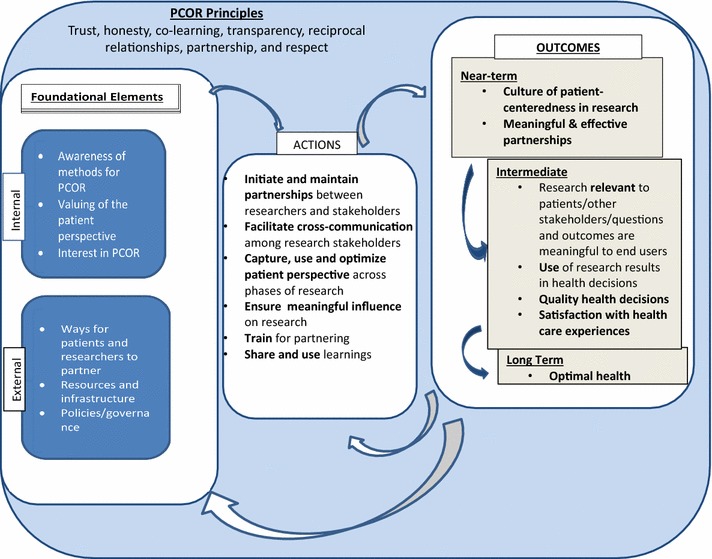
Conceptual model of patient-centered outcomes research

In formulating the model, we consulted the literature on CER, patient-centeredness, and patient engagement in research [8–15] and we worked with members of the PCORI Patient Engagement Advisory Panel on the range of concepts covered by the conceptual model and the relationships between them. This model has roots in CBPR, in early definitions of outcomes research [16], and in the tenets of patient-reported outcomes assessment [17, 18]. See the Fig 1.

The model is organized around three concepts: (1) *foundational elements* required for PCOR; (2) *actions* or behaviors involved in conducting PCOR; and (3) *outcomes* the results of actions using the foundational elements. *Principles* provide the ethical backdrop for PCOR. The extent to which research teams demonstrate the principles can be operationalized and measured. Their presence in this model is based on the premise that these principles must be demonstrated for the research to be PCOR. The extent to which research partnerships embody the principles can be positively and negatively impacted by the concepts in the model; that is, some principles like trust may be increased over time through positive experiences during the research process.

The six foundational elements in the model are divided into “internal” and “external” elements. The first three internal elements start with awareness of PCOR principles and methods. Patient-centered outcomes research partners are expected to be motivated by specific reasons, which requires knowledge of aspects of PCOR—what it is, why it is a worthwhile model of research to pursue, and how obtain and use patient perspectives. Research team members participating in PCOR must also value the patient perspective in research and believe there are benefits, whether ethical or practical or both, to capturing and using the patient perspective in research. A positive interest in PCOR is called out separately since awareness of PCOR practices and value for the patient perspective do not necessarily result in an interest in pursuing PCOR models of research.

Against these three internal factors are three external foundational elements. First, there must be a means for patients and researchers to communicate as research partners. These “channels” for interaction can be direct face-to-face meetings, email and online sharing, or other more structured forums for permitting these discussions to happen. There must be means to support that engagement, noted here as resources and infrastructure, including financial resources to ensure appropriate involvement of target communities in the research, and organizational structures to facilitate the conduct of PCOR. Finally, there must be permissive or at a minimum, non-obstructive organizational policies and governance which enable the implementation of the foundational elements.

Action elements represent engagement in research. They are (1) research partnership formation and maintenance between researchers and stakeholders; (2) multi-directional cross-communication rather than uni-directional communication, among all research stakeholders: patients, researchers, and other stakeholders; (3) the collection and use of the patient perspective across phases of research (e.g., hypothesis generation, study design, selection of outcomes, data analysis, dissemination); (4) checks for ongoing meaningfulness of partner influence on research, for example evidence that influence of patient and other stakeholder partners is present, and influence on the research extends beyond scientist members of the research team; and (5) training for partnering in research to enable both researchers and stakeholder partners to understand their roles and to have the knowledge necessary to fulfill those roles; and (6) sharing and use of learnings throughout the research process.

The outcomes of PCOR are organized into near-term, intermediate, and longer-term outcomes, as each of these types of outcomes are qualitatively distinct. The ultimate goal of PCOR is optimized health outcomes. Outcomes are hypothesized to feed back, both positively and negatively, to actions and foundational elements.

In the near term, hypothesized outcomes include (1) a culture accepting of PCOR along with (2) partnerships that are effective, in which partners can identify positive influence of each partner type. Following realization of near-term outcomes, PCOR should result in (1) research relevant to patients/other stakeholders; (2) improved use of research results in health decisions by all users; (3) improved quality of health decisions—enhanced by the research; and (4) improved satisfaction with health care experiences.

As described above the model includes principles for PCOR: trust, honesty, co-learning, transparency, reciprocal relationships, partnership, and respect [8, 11]. While additional principles for patient-centeredness in research have been suggested [9, 11], we limited the conceptual model principles here to those we thought are necessary to success of PCOR work. Using this model as the basis, future empirical research is needed on how to operationalize the principles, whether all principles are required for successful PCOR, and whether additional principles emerge as elements of successful PCOR.

The concept of engaged clinical research promoted by PCORI has many similarities with the concept of CBPR, with some key differences. CBPR has been defined as “a collaborative research approach that is designed to ensure and establish structures for participation by communities affected by the issue being studied, representatives of organizations, and researchers in all aspects of the research process to improve health and well-being through taking action, including social change,” [1, p. 25]. Like CBPR, PCOR is defined in part by active involvement of relevant community members beyond researchers. However, the PCORI definition of PCOR emphasizes importance of questions and outcomes to patients and informing health care decisions (see outcomes in model), an emphasis not found within all CBPR models. Also, although both CBPR and PCOR involve a “collaborative research approach,” the active social change orientation is not a requirement of PCOR.

The PCOR model shares valuing of the patient perspective with the PRO research of the last two decades [e.g., 17]. Capturing the patient voice is fundamental to PCOR as it is to both CBPR and PRO research. In contrast to PRO research, PCOR requires not just capture of patient voice with patients as research subjects, but inclusion of patient direction in the actual planning and conduct of the research. Both PRO research and PCOR address what is important to the patient, and subsequent questions and research actions derive from the patient view. PCOR adds to this the capture of patient and other stakeholder input on *how* the research is conducted, a dimension not represented in PRO research. The influence of patients not just on content but on methods used to collect information, strategies for subject recruitment, and strategies for dissemination expands the PCOR model well beyond methods of PRO research.

## Learning about engagement in research from stakeholders

PCORI is collecting information about research engagement and CER in multiple ways. The model described above and a companion evaluative model of engagement [19] guide this work, with PCORI’s evaluation framework providing substantial direction (http://www.pcori.org/blog/evaluating-the-pcori-way-building-our-evaluation-framework/). Below we describe some of the work underway.

To understand the public’s attitudes toward CER and engagement in research, patients with chronic health conditions, patients with rare diseases, and primary care clinicians including physicians, physician assistants, and nurses were recruited from an opt-in set of online panels. Full details of the survey are reported elsewhere [20, 21]. Of interest was the extremely limited familiarity among patients and clinicians of engaged research, patients, and other stakeholders working as partners in clinical research. Once provided with a definition of engaged research, both groups expressed interest in participating in this type of health research. Also of note, in this sample, few primary care clinicians were familiar with the term CER but once provided with a definition they endorsed the value of CER to clinical treatment decision making. Additional survey data are being collected now from broader samples of patients, clinicians, and researchers to further inform PCORI work.

## Learning about engagement from awardees

The first PCORI funding awards were announced in May 2012 when PCORI-funded 50 pilot projects to advance patient-centered outcomes research methods. Early in the projects, PCORI asked awardees to answer questions about their engagement with patients and other stakeholders in their projects; 47 (94 %) of the 50 awardees responded. Questions addressed types of stakeholders engaged, the stages and levels of engagement, an assessment of facilitators of and challenges to engaged research, and contributions of engaged stakeholders.

The majority of responding awardees (83 %) reported having engaged at least one patient or other stakeholder in the research by the time they completed the data collection tool; among those projects, respondents most commonly reported engaging patients/consumers (90 %), and clinicians (87 %). Engagement of clinic or health system representatives was reported by 44 %.

Awardees provided substantial free text responses, adding detail to the closed-ended questions. The main themes regarding initial contributions to the research projects from engaged stakeholders include changes to project methods, outcomes or goals, modifications to interventions, improvement of measurement tools and data collection methods, and interpretation of qualitative data. As one investigator noted, “I can say with confidence that our project (the methods and even the project goals) have evolved, in some cases dramatically, based on our collaborations with stakeholders.” Another respondent commented on the importance of genuine relationships: “participation was enhanced because they quickly realized that their role was not symbolic in nature but was integral to the project’s development in many ways.” Learnings about engagement and the perceived impact of engagement, from both researchers and stakeholder partners, will be collected at the end of these projects. Data collection about engagement for the rest of PCORI’s awardees, including both the researchers and stakeholder partners, is underway.

## Learning from engagement events

Since its inception, PCORI has held workshops across the nation to facilitate partnerships and to address the interests of patients, patient advocacy groups, and other healthcare stakeholders. PCORI surveys participants at the conclusion of each workshop event and then again 6 months later, asking specifically about further development of PCOR capabilities among attendees. To date, nearly 200 event participants have responded to the 6-month follow-up survey with a 42 % response rate. Of those respondents, over 86 % indicated that they had done something new to conduct, promote, or use patient-centered research after attending the PCORI event, including educating others, engaging patients in new ways in research initiatives, creating or joining a council to promote PCOR, and forming or joining a new research team or project using patient-centered approaches. A quarter of respondents indicated that they acted as a primary or co-investigator on a PCORI application. PCORI continues to track links between outreach events and subsequent PCOR activities.

## Advisory panel on patient engagement

PCORI is committed to integrating the patient and stakeholder perspective throughout its work. The legislatively mandated Advisory Panels include patients and other stakeholders, and an additional Advisory Panel was convened, the Advisory Panel on Patient Engagement, to ensure the highest patient engagement standards and a culture of patient-centeredness in all aspects of PCORI’s work and the research that we fund. The panel advises PCORI on programmatic initiatives, organizational evaluation strategies, and interdepartmental programs. As noted above, this Panel advised PCORI on the development of a conceptual model for PCOR [22] and provided recommendations to PCORI on the development of an organizational evaluation framework (see also (http://www.pcori.org/blog/evaluating-the-pcori-way-building-our-evaluation-framework/).

## Engagement in merit review

While individuals who may not have specific research methods training are not usually part of funding application review, PCORI recognizes the value of inclusion of perspectives of end users of research as the research applications are evaluated and includes patients and other stakeholders along with scientists in the review of funding applications. Scientist reviewers are required to score applications for all five of PCORI’s merit review criteria. Other reviewers are required to score three of those criteria.

In recognition of the newness of this type of stakeholder engagement, and to support high-quality reviews by those new to research application evaluation, PCORI has created a reviewer mentor program, in which each patient and other stakeholder reviewer is paired with a mentor experienced with PCORI merit review. The mentor provides ad hoc support, helping to explain PCORI’s criteria and how to apply them to application evaluation, and provides early guidance on application critiques.

PCORI analyzes use of merit review criteria by reviewer type and examines score changes before and after the reviewer group discussion of applications, as one way to quantify the impact of combining different perspectives in merit review. An analysis of merit review scoring data from the initial PCORI funding cycle demonstrated convergence of scores between researchers and other stakeholder reviewers, from pre-panel scores provided independently by application reviewers to scores entered following the in person-panel discussion [23]. Patient and other stakeholder scores changed more than did scientist reviewer scores from pre- to post-panel. Examination of scores from each cycle continues.

## Engagement rubric

PCORI has developed an Engagement Rubric as a tool to guide researchers in engaging patients and stakeholders in research and to highlight promising models of meaningful engagement. The Rubric includes the six principles of engagement in PCOR represented in the conceptual model: reciprocal relationships, co-learning, partnership, trust, transparency, and honesty [9, 24]. The Rubric is not intended to be comprehensive, prescriptive, or final. Instead, it provides a foundation for describing engagement and ultimately evaluating the impact of engagement.

## Supporting engagement through capacity building

One of PCORI’s strategic goals is to enhance the capabilities for conducting PCOR among patients, clinicians, researchers, and other stakeholders. To help overcome barriers to research engagement, PCORI offers “Pipeline to Proposal” awards, to help recipients build relationships with other individuals and groups interested in their health issue or topic of concern, create a strategy and tools to connect to potential research partners, and to develop governance structures and strategic plans for their budding communities around a research topic. These awards are intended to develop communities capable of identifying and refining a comparative effectiveness research question.

The PCORI Ambassador Program is another initiative supporting PCORI’s strategic goal of enhancing capabilities for conducting PCOR. The Ambassador initiative equips, trains, connects, and mobilizes patients, organizations, and other stakeholders to share PCORI’s vision and mission and PCOR principles with their respective communities, participate as full partners in research, and help ensure the sharing and use of information generated from PCORI-funded projects. As of October 1, 2014, PCORI has trained 82 Ambassadors across a variety of stakeholder communities, including: patients and caregivers, patient and caregiver advocates, researchers, clinicians, representatives from hospitals and health systems, purchasers, payers, industry, and policy makers. Ambassadors represent states from every region of the USA. The long-term goal is to have Ambassadors from every community across the healthcare system in every state, extending the reach of our engagement efforts and expanding knowledge about and participation in PCOR across the country.

## Infrastructure building and engagement: PCORnet

The National Patient-Centered Clinical Research Network, also known as PCORnet, was created by PCORI to improve the nation’s capacity to conduct comparative effectiveness research. This national resource aims to create a highly representative, interoperable, highly efficient “network of networks” that combines both electronic health records and patient-generated data to capture the full patient experience. In December 2013, PCORI-funded 29 network partners, 11 Clinical Data Research Networks (CDRNs; see Table 1) and 18 Patient-Powered Research Networks (PPRNs; see Table 2). CDRNs are health system-based networks, such as networks of academic medical centers, hospitals and physician practices. The PPRNs are a unique aspect of PCORnet, networks led and operated by patients, advocacy organizations, and clinical research partners who are interested in moving the research agenda forward for a specific medical condition. The heterogeneity of the 29 networks, and their complementary strengths, will provide a rich national resource for future comparative effectiveness research [25].

**Table 1:** PCORnet clinical data research networks

Clinical data research network organization (network name)	Organization type(s)
Accelerating Data Value Across a National Community Health Center Network (“ADVANCE”)	Network of low-income clinics
Chicago Community Trust (“CAPriCORN”)	Community trust large urban population
Children’s Hospital of Philadelphia (“PEDSNet”)	Children’s Hospitals Consortium
Harvard (“SCIHLS”)	Academic Medical Center
Louisiana Public Health Institute (“Louisiana Clinical Data Research Network—LACDRN”)	Health Information Exchange-based
Patient Outcomes Research To Advance Learning (“PORTAL”)	Integrated health systems
PaTH: Towards a Learning Health System in the Mid-Atlantic Region (“PaTH”)	Academic Medical Center
University of California San Diego (“pSCANNER”)	Academic Medical Center (Scanner) + VA
University of Kansas Medical Center (“Great Plains Collaborative”)	Academic Medical Center (CTSA)
Vanderbilt University (“Mid-South CDRN”)	Academic Medical Center
Weill Medical College (“NYC-CDRN”)	Community trust large urban population

**Table 2:** PCORnet patient-powered research networks

Patient-powered research network	Organization(s)	Condition(s)
Multiple Sclerosis Patient-Powered Research Network	Accelerated Cure Project for Multiple Sclerosis, Feinstein Kean, Life Data Systems	Multiple sclerosis
Sleep-Apnea-Patient-Centered Outcomes Network (SA-PCON) PPRN	American Sleep Apnea Association, Brigham and Women’s Hospital, Group Health, Morehouse Medical School, NYU and Columbia, ResMed, Sleep Research Network	Sleep apnea
ImproveCareNow: A Learning Health System for Children with Crohn’s Disease or Ulcerative Colitis	Cincinnati Children’s Hospital Medical Center, ICN Registry	Pediatric inflammatory bowel disease
The COPD Patient-Powered Research Network	COPD Foundation, CONCERT, COPD GENE	Chronic obstructive pulmonary disease
CCFA Partners Patient-Powered Research Network	Crohn’s and Colitis Foundation of America, Chronology, Patients Know Best, Validic, University of North Carolina	Inflammatory bowel disease
Arthritis Patient Partnership with Comparative Effectiveness Researchers (AR-PoWER PPRN)	Global Healthy Living Foundation, University of Alabama CERTS, Creakyjoints, CORRONA, American College of Rheumatology	Inflammatory arthritis
Mood Patient-Powered Research Network	Massachusetts General Hospital, Partners Health Care System	Major depressive disorder and bipolar disorder
The Health eHeart Alliance	University of California, San Francisco, American Heart Association	Cardiovascular health
American BRCA Outcomes and Utilization of Testing Patient-Powered Research Network (ABOUT Network)	University of South Florida, FORCE, Michigan Dept. of Community Health, Genomics, and Genetic Disorders	Hereditary breast and ovarian cancer
ALD Connect,	ALD Connect, Inc, Kennedy-Krieger Institute at JHU, Massachusetts General Hospital, Stanford University, University of Minnesota, University of Utah	X-linked adrenoleukodystrophy
NephCure Kidney Network for Patients with Nephrotic Syndrome	Arbor Research Collaborative for Health, NephCure Foundation	Primary Nephrotic Syndrome, Focal Segmental Glomerulosclerosis, Minimal Change Disease, Membranous Nephropathy Multiple Sclerosis
Patients, Advocates and Rheumatology Teams Network for Research and Service (PARTNERS) Consortium	Duke University, Arthritis Foundation, Childhood Arthritis & Rheumatology Research Alliance (CARRA), Friends of CARRA, Lupus Foundation of America, Pediatric Rheumatology Care & Outcomes Improvement Network (PR-COIN)	Juvenile Rheumatic Disease
Rare Epilepsy Network (REN)	Epilepsy Foundation, Columbia University, Research Triangle Institute	Aicardi Syndrome, Lennox-Gastaut Syndrome, Phelan-McDermid Syndrome, Hypothalamic Hamartoma, Dravet Syndrome, Tuberous Sclerosis
Community Engaged Network for All (CENA)	Genetic Alliance, Inc, University of California Davis, University of California San Francisco	Alstrom syndrome, Dyskeratosis congenital, Gaucher disease, Hepatitis, Inflammatory breast cancer, Joubert syndrome, Klinefelter syndrome and associated conditions, Psoriasis, Metachromatic leukodystrophy, Pseudoxanthoma elasticum
Patient Research Connection: PI-Connect	Immune Deficiency Foundation, Chronic Granulomatous Disease Association, SCID Angels for Life, Foundation Wiskott-Aldrich Foundation	Primary immunodeficiency disease
The DuchenneConnect Patient-Report Registry Infrastructure Project	Parent Project Muscular Dystrophy, Patient Crossroads, Geisinger Health Systems, UCLA	Duchenne and becker muscular dystrophy
Phelan-McDermid Syndrome Data Network	Phelan-McDermod Syndrome foundation, Epilepsy Foundation, CMBI-Harvard Medical School	Phelan-McDermid syndrome
The Vasculitis Patient-Powered Research Network	University of Pennsylvania, U of South Florida, Vasculitis Clinical Research Consortium, Vasculitis Foundation	Vasculitis

In addition to its size and scope, PCORnet is unique in its emphasis on engagement, specifically broader participation of all key stakeholders including patients, clinicians, health systems leaders, and payers. Patients have substantive roles in designing and planning of the infrastructure including the governance and use of the data, defining the research questions and identifying optimal ways of disseminating results. With increased stakeholder input into the network building capacity, PCORnet has the potential to support research questions that are more meaningful and relevant to patients.

Because of the diversity and size of the networks, PCORnet offers a unique opportunity to observe a wide range of engagement practices in network capacity building. At the end of the 18-month funding period, PCORI will have collected a large body of evidence on patient and stakeholder engagement in infrastructure development that will inform our current understanding of engagement challenges and promising practices.

## Generating evidence for engagement: PCORI’s “improving methods” portfolio

PCORI’s “Improving Methods for Conducting Patient-Centered Outcomes Research” program—one of PCORI’s five National Priorities for Research—is building a research portfolio to address the methodological gaps in the field of patient-centered CER, including the area of patient and stakeholder engagement. The portfolio includes projects that identify optimal methods for engaging patients and other stakeholders in the research process. One currently funded study^1^ is investigating how to reach and engage minority patients and stakeholders in research. Another^2^ aims at engaging patients in diverse communities in translating evidence-based guidelines into language that resonates with their communities.

PCORI also funds quantitative and qualitative data on *the impact of engagement on research*, including the value of patient-recommended outcomes for advancing knowledge of research topics, the speed of dissemination of research results, and the speed and comprehensiveness of uptake of relevant research findings into clinical practice. One awardee group^3^ is examining whether Community Review Boards (CRBs) represent an effective method of obtaining patient stakeholder input and whether CRB input results in research that is more patient-centered. Another project^4^ is assessing the impact of patient and stakeholder engagement on the development of patient decision aids.

In funding such research, PCORI recognizes the imperative for building an empiric evidence base for its engagement activities. This includes funding not only research on “how” to do engagement, but funding research that rigorously evaluates the impact of engagement on research design, conduct, analysis, and dissemination.

## Conclusion

PCORI requires engagement of patients or other healthcare stakeholders in the patient-centered CER it funds but engaging stakeholders in the work of PCORI extends far beyond the funded research. Patients and other stakeholders join scientists in evaluation of funding applications; they formally advise PCORI activities through the Advisory Panels; they are the focus of capacity building in the “Pipeline to Proposal” awards; and they are an integral part of PCORnet, the large national initiative to build CER infrastructure.

The conceptual model of PCOR presented here is intended to identify required elements for PCOR, provide a way to describe patient-centeredness in research, and provides a basis for evaluating the quality of engagement in patient-centered research. While growing interest in research engagement has led to engagement-specific frameworks and definitions, no single conceptual model has yet connected enabling elements to specific research-related actions and to intended research outcomes. The link between PCOR and improved health decisions and health outcomes is assumed but has yet to be tested. The model presented here can aid with future empirical evaluations of the link between elements of PCOR and the ultimate goals of PCOR.

As the evidence on impacts of engagement in research accumulates, PCORI will continue its model of evaluating not only the research it funds but also engagement in research prioritization, merit review, and infrastructure building. Through evaluation activities as well as through research funding PCORI continues to add to the evidence base on engagement in research.

## Footnotes

1 Barbara Turner (PI), “Evaluating Methods to Engage Minority Patients and Caregivers as Stakeholders”.

2 Don Neuse (PI), “Creating Locally Relevant Health Solutions with the Appreciative Inquiry and Boot Camp Translation Method”.

3 Consuelo Wilkins (PI), “Improving Patient Engagement and Understanding Its Impact on Research through Community Review Boards”.

4 Holly Witteman (PI), “Applying Methods of User-Centered Design to Achieve Patient-Centered Care”.
